# Clinical Outcomes of Individualized Electrostimulation Using a Wearable Electro Suit and Qualitative Feedback From a Mixed Cohort of Survivors of Stroke and Spinal Cord Injury With Spasticity: Case Series

**DOI:** 10.2196/81522

**Published:** 2026-06-26

**Authors:** Jia Min Yen, Nur Shafawati Kamsani, Hwa Sen Lai, Ning Tang, Effie Chew

**Affiliations:** 1 Division of Rehabilitation Medicine, Department of Medicine, National University Hospital, 5 Lower Kent Ridge Road, Singapore, 119074, Singapore, 1 67795555; 2 Rehabilitation Medicine, Department of Medicine, Alexandra Hospital, Singapore, Singapore; 3 Department of Rehabilitation, Alexandra Hospital, Singapore, Singapore

**Keywords:** electrostimulation, Mollii Suit, assistive technology, spasticity, gait

## Abstract

**Background:**

Various forms of electrical stimulation have been integrated into the multimodal management of spasticity. However, high-frequency electrical stimulation can potentially induce muscle fatigue. The Exopulse Mollii Suit (EMS) is a multichannel full-body garment that delivers low-frequency (20 Hz), low-amplitude (20 V), subthreshold sensory stimulation aimed at reducing spasticity.

**Objective:**

Primarily, we examined the effects of a single session of the EMS on spasticity in 7 participants with chronic stroke or cervical spinal cord injury (SCI), specifically those with upper or lower limb spasticity impacting function and gait who were able to walk with minimal or no assistance (Functional Ambulatory Category scores of 2‐5). We assessed the impact on gait and ambulatory function, as well as user perceptions of usability and acceptability.

**Methods:**

Participants wore the EMS for 60 minutes, consisting of 30 minutes of standardized goal-directed activities performed in two 15-minute blocks, interspersed with 15-minute rest breaks. Measurements included the Modified Tardieu Scale with surface electromyography for spasticity and functional mobility tests (Functional Ambulatory Category, 10-meter walk test, 5 times sit-to-stand test, and step test). Spatiotemporal gait parameters were quantified using a markerless vision-based motion capture system using the OpenPose BODY25 pose estimation model.

**Results:**

On the basis of the Modified Tardieu Scale and surface electromyography signals, improvements in spasticity were only observed in 2 participants. However, 4 participants demonstrated faster walking speeds. Improvements in the 5 times sit-to-stand test and step test were noted in 3 and 4 participants, respectively. Spatiotemporal gait parameters revealed improvements in gait symmetry in 6 participants. Qualitative feedback based on the Assistive Technology Usability Questionnaire for People With Neurological Diseases (NATU Quest) returned positive results in 3 participants. Overall outcomes, defined as meeting the individualized goals of each participant, were positive in 4 participants.

**Conclusions:**

This case series provides preliminary evidence that a single session with the EMS may offer benefits for functional mobility and gait quality for individuals with spasticity resulting from stroke or SCI. To our knowledge, this is the first report examining the effects of the EMS in participants with SCI and the first to include spatiotemporal gait parameters associated with its use. However, the small sample size, variable outcomes, and lack of a control group necessitate caution in interpreting these findings and preclude definitive conclusions regarding the efficacy of the EMS. Larger, controlled trials with repeated sessions of EMS use are required to establish the effectiveness and optimal application of the EMS for spasticity management.

## Introduction

Spasticity is a common and disabling complication of central neurological diseases, particularly following stroke [1] and spinal cord injury (SCI) [2]. It is characterized by a velocity-dependent increase in tonic stretch reflexes [3] and involuntary muscle activation [4], which disrupt motor control. When synchronized agonist and antagonist muscle activity is disturbed, fluid movement is no longer possible. Consequently, the ability to perform activities of daily living, including transfers and ambulation, is significantly impaired.

Given the profound impact of spasticity on function and quality of life, effective management is crucial during rehabilitation to restore mobility—a top priority following neurological injury [5]. Spasticity treatments have largely focused on oral pharmacological agents and interventions, such as botulinum toxin injections, alcohol neurolysis, or intrathecal baclofen, as nonpharmacological interventions have limited efficacy in isolation [6-8]. While pharmacological treatments may pose a risk of systemic side effects, such as drowsiness, risks associated with physical modalities, such as electrical stimulation are generally minimal when used appropriately. Hence, various forms of electrical stimulation have been integrated into the multimodal approach to the management of spasticity [9]. Among these, neuromuscular electrical stimulation (NMES) using 0 to 100 Hz biphasic symmetrical waveform electrical stimulation [10] and transcutaneous electrical nerve stimulation (TENS) using high frequencies of more than 100 Hz [11] have been shown to be effective in reducing spasticity. Their efficacy in reducing spasticity is further enhanced when combined with physical therapy interventions, such as range of motion and functional task training [12]. The proposed mechanisms include increased presynaptic inhibition of hyperactive stretch reflexes, a reduction in neural circuitry hyperexcitability, and a decrease in cocontraction of spastic antagonists by the use of TENS [13,14]. In addition, NMES has been shown to improve walking dysfunction in stroke survivors, leading to improved gait speed and balance [15]. However, the potential limitation of high-frequency electrical stimulation is the induction of muscle fatigue [16], which may in turn exacerbate spasticity [17].

The Exopulse Mollii Suit (EMS; Inerventions AB) is a multichannel full-body garment (jacket and pants) consisting of 58 electrodes. It is powered by a 4×AAA battery-powered control unit that transmits low-energy electrical pulses to the garments via the connectors. The suit comes in 37 sizes, starting from 104 cm up to 5XL for women and men. In contrast to NMES and TENS, the EMS uses 2 mA monophasic square waves to deliver low-frequency (20 Hz), low-amplitude (20 V) electrical stimulation through prepositioned electrodes to stimulate up to 40 key muscle groups simultaneously. Its pulse width is adjustable from 30 to 175 microseconds, with a period length of 50 milliseconds between pulses, to allow for varying intensities of electrical stimulation. During stimulation, the pulse width increases by 5 microseconds every 5 seconds from 25 microseconds until the preset pulse width is reached. This targeted suprasensory and submotor electrical stimulation aims to modulate the stretch reflex and mitigate spasticity via reciprocal inhibition of antagonistic muscles mediated by Renshaw cells [18,19]. Apart from being noninvasive and nonpharmacological, the EMS may reduce the risk of muscle fatigue associated with higher-frequency stimulation and can potentially be self-administered at home, making it a compelling option for improving function with minimal adverse effects.

In this study, we aimed primarily to examine the acute effects of individualized electrostimulation using the EMS on spasticity in participants with chronic stroke or SCI. Secondarily, we aimed to assess the impact of a single session on gait and ambulatory function. We also assessed user perceptions of the usability and acceptability of the EMS.

## Methods

### Study Design and Participants

This was a case series of participants with spasticity from chronic stroke or SCI. Participants were included if they had upper or lower limb spasticity that impacted function and gait and were able to walk with minimal or no assistance (ie, Functional Ambulatory Category [FAC] scores of 2‐5) [20]. Exclusion criteria included preexisting medical conditions preventing safe use of the EMS, such as the presence of implanted electromagnetic devices (eg, shunts, pacemakers, and intrathecal baclofen pumps), epilepsy, malignancy, uncontrolled cardiac conditions, and poor skin condition. Patients with recent initiation of oral muscle relaxants or botulinum toxin injections in the past 6 months were also excluded. A convenience sample was identified by the study team and contacted by the principal investigator for participation.

### Study Protocol

An interview for goal selection was performed for each participant before the study. All outcome measures were performed before and after a single session of stimulation with the suit.

**Figure 1.**
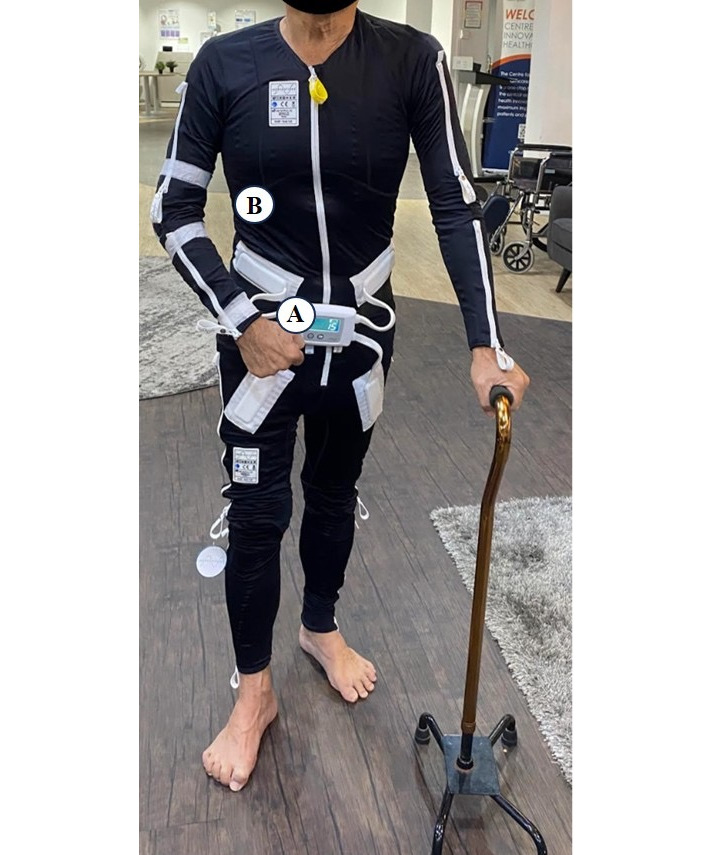
Participant walking with a broad-based quad stick while wearing the EMS. (A) Individualized stimulation parameters are programmed into the control unit that generates electrical pulses. (B) Jacket and pants containing 58 embedded electrodes in direct contact with the skin delivering electrical stimulation.

**Figure 2.**
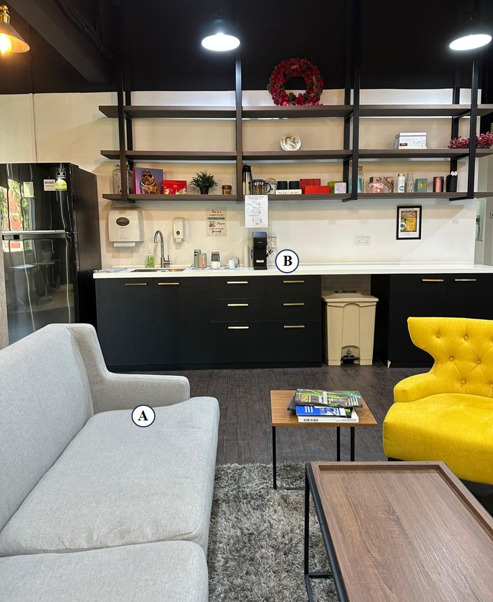
Living room–like setting where the study was conducted. (A) Participant could sit on the sofa to rest or read, simulating a home environment. (B) Activities of daily living, such as preparing a drink, could be performed in this area.

Participants were fitted with appropriately sized suits to ensure good contact between the electrodes and the skin. They donned and doffed the EMS with assistance, ensuring that it was correctly worn—for example, zippers were positioned on the outside of the forearm, forming a straight line between the elbow and the little finger, and the seam was placed above the knee. Stimulation parameters were determined by an experienced physical therapist from Exopulse based on the results of the initial clinical assessment. The stimulation parameters were individualized and set to stimulate the antagonists of spastic muscles. A preset program was used to adjust the intensity of electrical stimulation across levels 6 to 22, depending on both the size of the target muscle group and severity of spasticity. Higher-frequency electrical stimulation is used for larger muscle groups or to manage more severe spasticity. Participants wore the suit for 60 minutes. This intervention period included 30 minutes of standardized goal-directed activities performed in two 15-minute blocks, interspersed with 15-minute rest breaks. These light activities simulated daily activities, such as food preparation or walking, and were goal directed. Figure 1 shows a participant walking with a broad-based quad stick while wearing the EMS. Figure 2 depicts the living room–like setting where the study was conducted.

### Study Outcomes

Outcome measures before and after stimulation included measures of spasticity, using the Modified Tardieu Scale (MTS) [21] paired with surface electromyography (sEMG). Assessments of mobility included FAC [20], 10-meter walk test (10MWT) [22] for walking speed, five times sit-to-stand test [23] for transfer skills, and step test [24] for dynamic balance were scored. All outcomes were assessed by an experienced physiotherapist not involved in determining the stimulation parameters for the participants.

sEMG signals were recorded during high-velocity passive stretch movements (V3) during MTS measurements using Cometa EMG system (version 8.7.6.0; Cometa SRL) via lightweight wireless electromyography electrodes placed on the muscle belly of a standardized set of target muscles. The sampling rate for sEMG was 2000 Hz. Raw sEMG data were then processed by (1) low-pass filtering at 500 Hz with an eighth-order Butterworth filter and high-pass filtering at 20 Hz with a ninth-order Butterworth filter, (2) full wave rectification, and (3) smoothing with the root mean square (RMS) method. The time domain RMS feature of the sEMG signals was extracted. Three indexes were used for spasticity measurement: (1) The RMS (sEMG) of the agonist signal, (2) the RMS (sEMG) of the antagonist signal, and (3) spasticity index [25]:


$$sEMG\;index={RMS}_{\text{agonist}}/\left({RMS}_{\text{agonist}}+{RMS}_{\text{antagonist}}\right)$$

For example, the *sEMG index* of biceps measured during passive elbow extension =RMSoftriceps/RMSoftriceps+RMSofbiceps, while the *sEMG index* of the gastrocnemius measured during passive knee flexion = RMSofgastrocnemius/RMSofrectusfemoris+RMSofgastrocnemius.

The spasticity angle derived from the MTS was corroborated with the sEMG index. A larger sEMG index indicates less spasticity.

Full-body sagittal plane videos of participants walking at comfortable walking speed, were recorded using an iPad Pro (Apple) at a height of approximately 1.5 m from the ground before and after use of the EMS. These were used for observational gait analysis, as well as quantification of spatiotemporal gait parameters using a markerless vision-based motion capture system. The system used OpenPose BODY25 pose estimation model [26] for 25-keypoint human pose estimation, gait events detection, and derivation of gait parameters via the coordinate-based treadmill algorithm method [27]. The system had previously been validated to detect gait abnormalities [28,29]. A single most accurately captured gait cycle from each walking trial was used to assess spatiotemporal parameters. Additionally, to quantify gait symmetry of participants with stroke, ratio index (RI) of step length was calculated [30].

RI=X1/X2, where X1 is the step length of left lower limb and X2 is the step length of the right lower limb [31]. In participants with stroke, the step length of the paretic lower limb is used as the numerator.

The Assistive Technology Usability Questionnaire for People With Neurological Diseases (NATU Quest) [32] was used to assess acceptability and perceived effectiveness of the EMS and was performed by the same study team member that performed the initial assessment. Scores range from 0 to 5, with 0 to 2 reflecting negative outcomes and 3 to 5 indicating positive ones. An average score of 3 indicates marginal usability, while scores of 4 or 5 denote high usability.

### Ethical Considerations

The study has been reviewed and approved by the Domain Specific Review Board, part of the National Healthcare Group (2023/00832) in Singapore.

## Results

### Demographics and Baseline Characteristics

Seven participants took part in the study. All participants were male due to recruitment constraints, which may limit generalizability. The characteristics of the participants, patient-defined goals, and goal-directed activities performed during the trial, are outlined in Table 1. Four participants had spastic hemiparesis due to stroke, while 3 had spastic tetraparesis due to cervical SCI. All patients had goals focused on improving function, either related to walking or performing activities of daily living.

**Table 1.** Demographics and characteristics of participants^a^.

ID	Age (y)	Diagnosis	Time from injury (m)	Motor impairments	Individualized goals	Goal-directed activities performed	FAC^b^ score	Walking aid	Clinical gait abnormality
1	68	Left lentiform nucleus bleed	12	Right spastic hemiparesis (MRC^c^ grade 4 proximally) with foot drop	Improve hand function, upper limb spasticity, and walking while reducing dependence on the ankle-foot orthosis	Maintaining grip on a cup and walking	3	Broad-based quad stick	Increased pelvic obliquity, decreased knee flexion, and foot drop with high steppage during swing phase
2	65	Left subcortical infarct	20	Right spastic hemiparesis (MRC grade 4 proximally and MRC grade 1 distally)	Increase confidence in walking	Sit-to-stand practice and walking	4	Walking stick	Decreased knee flexion and ankle dorsiflexion during swing phase
3	61	Left thalamic bleed	31	Right spastic hemiparesis (MRC grade 4) with ataxia	Improve walking distance and the ability to perform daily activities	Upper limb activities such as stacking cones and blocks, using a mobile phone, and walking	3	Walking stick	Knee hyperextension during stance phase
4	48	Right subcortical infarct	5	Left spastic hemiparesis (MRC grade 4)	Improve hand function and walking speed	Upper limb activities, including preparing a drink, stacking cones and blocks, and walking	5	Nil	Decreased knee flexion and ankle dorsiflexion during swing phase
5	56	AIS^d^ C C5 spinal cord injury	350	Spastic paraparesis	Improve walking and balance	Reading a book, sit-to-stand practice and walking	3	Walking stick	Crouched gait with increased hip adduction and anterior pelvic tilt; decreased hip extension during stance phase and decreased knee extension during swing phase
6	76	AIS C C5 spinal cord injury	50	Spastic tetraparesis	Improve hand dexterity, lower limb spasticity and walking endurance	Upper limb activities including stacking cones and blocks, sit-to-stand practice, and walking	3	Nil	Decreased right hip flexion, knee flexion, and ankle dorsiflexion during swing phase
7	27	AIS D C6 spinal cord injury	30	Spastic tetraparesis	Improve walking speed and endurance	Walking	3	Nil	Increased hip adduction; anterior pelvic tilt; decreased knee extension on swing and stance phase bilaterally, worse on right lower limb; increased ankle plantarflexion on stance phase on right lower limb

a All participants were male.

b FAC: Functional Ambulatory Category.

c MRC: Medical Research Council.

d AIS: American Spinal Injury Association Impairment Scale.

### Primary Outcome: Spasticity

Only 1 participant with stroke and 1 participant with SCI demonstrated improvement in MTS greater than minimal detectable change of 17.16° [33] (Table 2). The sEMG index corroborated the reduction in spasticity in these muscles (Multimedia Appendix 1).

**Table 2.** Pre- and postintervention Modified Tardieu Scale (MTS) measurements.

Participant		Muscle	Before MTS, R2-R1^a^ (degrees)	After MTS, R2-R1(degrees)	Change (degrees)	Before MTS, X^b^	After MTS, X	Change in X
Stroke								
	1	Right biceps brachii	57	86	29	2	2	0
	1	Right gastrocnemius	27	31	4	2	2	0
	2	Right biceps brachii	35	38	3	2	2	0
	2	Right gastrocnemius	30	22	−8	3	3	0
	3	Right biceps brachii	38	35	−3	2	2	0
	3	Right gastrocnemius	30	45	15	2	2	0
	4	Left biceps brachii	13	0	−13	2	0	−2
	4	Left triceps brachii	93	0	−93^c^	2	0	−2
	4	Left rectus femoris	35	40	5	2	2	0
	4	Left gastrocnemius	30	25	−5	2	2	0
Spinal cord injury								
	5	Right rectus femoris	17	40	23	2	2	0
	5	Right gastrocnemius	0	36	36	0	2	2
	5	Left rectus femoris	13	41	28	2	2	0
	5	Left gastrocnemius	0	30	30	0	2	2
	6	Right biceps brachii	58	0	−58^c^	2	0	−2
	6	Right rectus femoris	20	45	25	2	2	0
	6	Left rectus femoris	28	15	−13	2	2	0
	7	Right rectus femoris	32	66	34	2	2	0
	7	Right tibialis anterior	40	28	−12	2	2	0

a R2: the angle of full range of motion, taken at a very slow speed (V1); R1: the angle of muscle reaction in which a catch or clonus is found during a quick stretch (V3).

b X: quality of muscle reaction score in the Modified Tardieu Scale.

c Greater than minimal detectable change.

### Secondary Outcomes: Function

#### Assessments of Mobility

FAC remains unchanged for all participants after the EMS. However, all participants with SCI and 1 participant with stroke demonstrated improved walking speed greater than SE of the measure of 0.05 m/s [34,35]. Two participants with SCI and 1 participant with stroke showed improvements reaching minimal clinically important difference in five times sit-to-stand test of 2.27 seconds and 1.94 seconds, respectively [36,37]. Furthermore, improvements in the step test were observed in 2 participants with SCI and 2 participants with stroke (Table 3). Data on upper limb function are presented in Multimedia Appendix 1.

**Table 3.** Mobility assessments before and after Exopulse Mollii Suit (EMS) use.

	FAC^a^ score		10MWT^b^ (m/s)			5xSTS^c^ (s)			Step test	
	Before EMS	After EMS	Before EMS	After EMS	Change (%)	Before EMS	After EMS	Change (%)	Before EMS	After EMS
Stroke										
Participant 1	3	3	0.19	0.19	0	22	27.68	25.82	4	5
Participant 2	4	4	0.31	0.25	−19.35	22	23.15	5.23	5	4
Participant 3	3	3	0.31	0.29	−6.45	62.85	58.75^d^	−6.52	5	4
Participant 4	5	5	0.77	0.84^d^	9.09	12.94	12.63	−2.40	10	14
Spinal cord injury										
Participant 5	3	3	0.24	0.35^d^	45.83	21.56	21.71	0.70	7	6
Participant 6	3	3	0.35	0.41^d^	17.14	34.88	22.72^d^	−34.86	5	9
Participant 7	3	3	0.36	0.57^d^	58.33	19.90	16.48^d^	−17.20	7	9

a FAC: Functional Ambulatory Category.

b 10MWT: 10-meter walk test.

c 5xSTS: five times sit-to-stand test.

d Greater than SE of the measure and minimal clinically important difference.

#### Spatiotemporal Gait Parameters

In participants with stroke, gait abnormalities were relatively unchanged after EMS use. However, gait symmetry (RI) improved in 3 of 4 patients with stroke. Single-support time increased for 2 participants and double-support time decreased in 1. Specifically, walking speed, cadence, and step time improved in participants 2 and 4 (Table 4) [38].

**Table 4.** Spatial and temporal gait parameters of participants with stroke before (pre) and after (post) using the Exopulse Mollii Suit.

	Participant 1^a^				Participant 2^b^				Participant 3^c^				Participant 4^d^			
	Left		Right (affected)		Left		Right (affected)		Left		Right (affected)		Left (affected)		Right	
	Pre	Post	Pre	Post	Pre	Post	Pre	Post	Pre	Post	Pre	Post	Pre	Post	Pre	Post
Spatial parameters (m)																
Step length	0.37	0.35	0.34	0.30	0.36	0.33	0.45	0.32	0.45	0.42	0.34	0.34	0.87	0.72	0.71	0.76^e^
Stride length	0.67	0.65	0.71	0.69	0.68	0.51	0.81	0.67	0.61	0.65	0.77	0.76	1.39	1.44^e^	1.58	1.48
Temporal parameters (s)																
Double support	1.91	1.97	1.72	2.03	0.94	0.68^e^	0.94	0.85^e^	0.67	0.71	0.37	0.67	0.43	0.45	0.40	0.44
Single support	0.78	0.77	0.56	0.60^e^	0.72	0.83^e^	0.46	0.48^e^	0.48	0.50^e^	0.62	0.37	0.48	0.41	0.58	0.49
Total stance time (% of total gait cycle)	—^f^	—	2.27 (70)	—2.64 (79)	—	—	1.48 (67)	—1.35 (64)	—	—	0.99 (76)	1.05 (70)	0.92 (61)	—0.86 (64)	—	—
Total swing time (% of total gait cycle)	—	—	0.97 (30)	—0.71 (21)	—	—	0.73 (33)	—0.75 (36)	—	—	0.31 (24)	0.45 (30)	0.59 (39)	—0.48 (36)	—	—
Walking speed (m/s)	0.20	0.20	0.22	0.20	0.32	0.25^e^	0.37	0.34^e^	0.39	0.41^e^	0.56	0.49	0.93	1.07^e^	1.10	1.10
Cadence (step/min)	36.94	36.47	37.03	35.22	56.72	58.33^e^	55.62	59.78^e^	77.45	76.23	83.27	77.87	80.03	88.93^e^	83.27	89.48^e^
Step time	1.20	1.17^e^	2.04	2.12	0.80	0.81	1.36	1.16^e^	0.72	0.62	0.77	0.92	0.80	0.75	0.64	0.59
Stride time	3.25	3.29	3.24	3.41	2.12	2.06	2.16	2.01^e^	1.55	1.57	1.44	1.54	1.50	1.35	1.44	1.34

a Ratio index (step length): baseline 0.92; after EMS 0.86.

b Ratio index (step length): baseline 1.25; after EMS 0.97.

c Ratio index (step length): baseline 0.76; after EMS 0.81.

d Ratio index (step length): baseline 1.23; after EMS 0.95.

e Greater than minimal detectable change.

f Total stance and swing time were only recorded for the stroke-affected side.

In participants with SCI, improved gait patterns were observed after use of the EMS. For instance, there was less hip adduction, increased hip flexion, and knee extension during the swing phase in participant 5. Spatial and temporal parameters also improved. There was a reduction in double-support time, an increase in single-support time, and longer step and stride lengths. Additionally, walking speed, cadence, step time, and stride time improved [39]. The stance-to-swing ratio also improved [40] (Table 5).

**Table 5.** Spatial and temporal gait parameters in participants with spinal cord injury before (pre) and after (post) using the Exopulse Mollii Suit.

	Participant 5^a^				Participant 6^b^				Participant 7^c^			
	Left		Right		Left		Right		Left		Right	
	Pre	Post	Pre	Post	Pre	Post	Pre	Post	Pre	Post	Pre	Post
Spatial parameters (m)												
Step length	0.37	0.47	0.44	0.47	0.44	0.41	0.09	0.25^d^	0.49	0.58	0.34	0.44
Stride length	0.73	0.87	0.87	0.80	0.49	0.66	0.58	0.69	0.77	1.03	0.83	1.12
Temporal parameters (s)												
Double support	1.89	0.99^d^	1.52	1.13	0.67	0.49	0.65	0.39	0.82	0.58	0.72	0.53
Single support	0.54	0.48	0.71	0.57	0.42	0.55	0.38	0.45	0.53	0.62	0.38	0.37
Total stance time (% of total gait cycle)	2.44 (81)	1.47 (75)	2.23 (80)	1.74 (76)	1.09 (73)	1.04 (66)	1.03 (71)	0.84 (64)	1.35 (77)	1.22 (76)	1.10 (70)	0.90 (63)
Total swing time (% of total gait cycle)	0.54 (19)	0.49 (25)	0.55 (20)	0.55 (24)	0.40 (27)	0.54(34)	0.42 (29)	0.47 (36)	0.40 (23)	0.39 (24)	0.47 (30)	0.53 (37)
Walking speed (m/s)	0.25	0.42^d^	0.30	0.36	0.34	0.45	0.36	0.50	0.44	0.63^d^	0.50	0.73^d^
Cadence (step/min)	40.58	58.56^d^	41.88	53.56	81.45	82.79	81.44	86.26	68.93	73.88	73.13	78.30
Step time (s)	1.37	1.04	1.45	0.99	0.70	0.67	0.77	0.77	0.78	0.74	0.86	0.88
Stride time (s)	2.96	2.05	2.87	2.24	1.47	1.45	1.47	1.39	1.74	1.62	1.64	1.53

a Ratio index (step length): baseline 0.90; after EMS 1.09.

b Ratio index (step length): baseline 4.89; after EMS 1.64.

c Ratio index (step length): baseline 1.44; after EMS 1.32.

d Greater than minimal detectable change.

#### Qualitative Feedback

**Figure 3.**
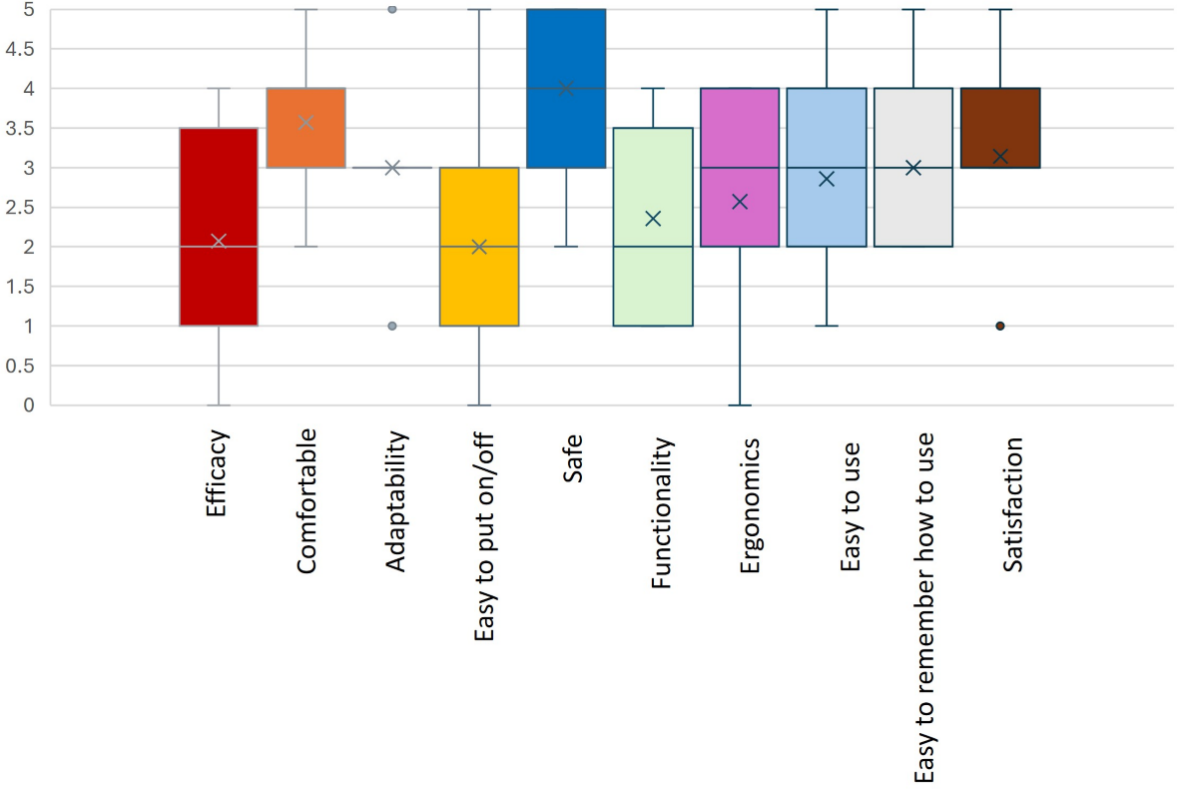
Qualitative feedback of Exopulse Mollii Suit.

The NATU Quest results are shown in Figure 3. On a Likert scale of 1 to 5, the EMS was scored in 10 distinct categories including efficacy, comfort, adaptability, ease of donning and doffing, safety, functionality, ergonomics, satisfaction, ease of use and remembering how to use. Overall, 3 of 7 users returned positive results, as defined by an average overall score of 3 or more. Median scores were positive in comfort, adaptability, safety, ease of remembering how to use, and satisfaction (Figure 3).

### Summary of Outcomes

An overall outcome was deemed positive if the EMS met the individualized goals specified in Table 1, and negative if these goals were not accomplished. Three participants with SCI and 1 participant with stroke had positive outcomes. Table 6 summarizes the quantitative outcomes and qualitative feedback on the EMS for each patient.

**Table 6.** Summary of key outcomes: quantitative and qualitative response to Exopulse Mollii suit (EMS) for each patient.

	Spasticity outcome	Gait outcome	Mobility outcome	Qualitative feedback	Overall outcome
Stroke					
Participant 1	Worsened spasticity angle and sEMG^a^ index in right biceps brachii	Increased single support time Decreased walking speed, cadence, and step length on stroke affected side	Improved ST^b^ and worsened 5xSTS^c^	Felt the EMS was comfortable and safe However, the overall usability score indicated notable challenges in usability	Negative
Participant 2	Equivocal	Improved gait symmetry, increased single support time and decreased double support time Increased walking speed on stroke affected side	Worsened 10MWT^d^ and ST	Felt the EMS was safe, easy to use and easy to remember how to use it However, the overall usability score indicated notable challenges in usability	Negative
Participant 3	Equivocal	Improved gait symmetry Increased double support time, decreased walking speed on stroke affected side	Improved 5xSTS and worsened ST	The overall usability score indicated notable challenges in usability	Negative
Participant 4	Improved spasticity angle and sEMG index in left triceps	Improved gait symmetry Increased walking speed and increased cadence on stroke affected side	Improved 10MWT and ST	Felt the EMS was comfortable and safe However, the overall usability score indicated notable challenges in usability	Positive; improved walking
Spinal cord injury					
Participant 5	Worsened spasticity angle and sEMG index in right and left rectus femoris	Improved gait symmetry Decreased double-support time Increased walking speed and cadence on left lower limb	Improved 10MWT and worsened ST	Felt the EMS was efficacious, comfortable, adaptable, easy to put on and off, safe, facilitates movement, ergonomic, easy to use, easy to remember how to use it, and is satisfied The usability score reflected a high level of usability	Positive; improved walking
Participant 6	Improved spasticity angle in right biceps but worsened spasticity angle and sEMG index in right rectus femoris	Improved gait symmetry	Improved 10MWT, 5xSTS, and ST	Felt the EMS was comfortable Overall, the usability score indicates marginal usability	Positive; improved walking and upper limb function
Participant 7	Equivocal	Improved gait symmetry Increased walking speed on both lower limbs	Improved 10MWT, 5xSTS, and ST	Felt the EMS was safe, ergonomic and is satisfied Overall, the usability score indicates marginal usability	Positive; improved walking

a sEMG: surface electromyography.

b ST: step test.

c 5xSTS: five times sit-to-stand test.

d 10MWT: 10-meter walk test.

## Discussion

### Principal Findings

Our study investigated the acute effects of individualized electrostimulation using a single session with the EMS on spasticity and ambulatory function in participants with stroke and SCI. While improvements in objective measures of spasticity at rest were modest, improvements in ambulatory function and gait parameters were observed.

Four of 7 (57.1%) participants improved in walking speed (10MWT), with only participants with SCI exceeding the minimal clinically important difference of 0.06 m/s [41-43]. The limited response in participants with stroke may be due to the responsiveness of the test, particularly in more severely impaired participants [44]. The limited dose of intervention, as well as other factors impacting on mobility including weakness, impaired muscle synergies, sensory impairment, and impaired endurance may also be contributory. Furthermore, participants who were dependent on physical assistance or had slower walking speed did not show improvements on 10MWT. Functional endurance also influences performance in 10MWT particularly when walking requires considerable effort [45,46]. Outcomes were measured immediately after the EMS session, when participants may have experienced fatigue from the standardized goal-directed activities. Nevertheless, improvements were noted in step test (4 of 7 participants), reflecting better dynamic balance during weight shift and paretic lower-extremity motor control; as well as step-length symmetry (n=6, 85.7%), reflecting increased energy efficiency during walking [47]. Furthermore, step test and step-length symmetry are both predictors of falls risk [48-50]. A trend toward increased single-support time and decreased double-support times in our cohort also suggests improved balance and neuromuscular control [51].

Our findings of modest improvement in spasticity measures (n=2, 28.6%) at rest, including MTS and sEMG indices, corroborated with a previous double-blind crossover study which compared the EMS at 2 different frequencies (20 Hz and 30 Hz) with sham stimulation in patients with chronic stroke and reported no observed reduction in spasticity [52]. In contrast, Palmcrantz et al [53] reported the effectiveness of repeated sessions with the EMS in decreasing wrist flexor spasticity in patients with chronic stroke in a home setting (60 minutes every second day for 6 weeks; total 21 sessions). These disparate findings highlight the need for further investigation into the dose-response relationship and the potential for cumulative effects with repeated EMS sessions. Participants within our cohort who had clinically significant improvements in MTS were also those with greater spasticity, as indicated by larger spasticity angles. Pennati et al [52] also reported high interindividual variability in response to the EMS and suggested that it might be more efficacious in severe spasticity.

The observed improvements in gait symmetry among participants with stroke (3/4, 75%) are encouraging, as they suggest the potential for enhanced walking efficiency. The more pronounced improvements in gait parameters among participants with SCI warrant further investigation into possible differences in the mechanisms of response to the EMS between stroke and SCI populations. These differences may relate to differences in spinal and supraspinal mechanisms of spasticity, as well as varying extent of involvement of the descending pathways responsible for spasticity and its subsequent adaptations [54]. Additionally, presynaptic inhibition of Ia afferent terminals in muscles may remain unchanged in patients with hemiplegic stroke, which could influence responsiveness to the EMS [55]. In this limited series, more participants with SCI reported a favorable response to the EMS compared with participants with stroke. To our knowledge, this is the first report of the effects of the EMS in participants with SCI.

Qualitative feedback was mixed, with a median rating of 2 for ease of donning and doffing, mirroring that of a previous structured interview in patients with stroke and cerebral palsy who had spasticity, who described difficulty in wearing the suit at home for self-administered electrotherapy [56]. This is not unexpected as the rubber electrodes used in the EMS may cause friction against the skin but are necessary to manage impedance and ensure effective electrostimulation [57]. Concerns regarding the ease of donning and doffing, ergonomics, and functionality highlight significant barriers to self-administration and adherence and should be addressed in future iterations of the device to support wider adoption for home use. However, this score should also be interpreted with caution as it reflects the initial impression with a single-session trial. Nevertheless, most participants expressed satisfaction with the session. Others have reported improved patient well-being and muscle-relaxing effect during stimulation [58].

### Limitations

We recognize that the absence of a control group and the small sample size in our study limit the reproducibility and generalizability of our findings. Future studies should also aim for greater gender representation. Observed gains in spasticity, gait, and mobility measures could have been confounded by practice effects or neuromuscular warm-up. These factors may directly confound mobility and gait outcomes and hence preclude definitive attribution of the observed changes to EMS use. We designed a structured and clearly defined protocol to standardize the duration of the EMS to 60 minutes for all patients, comprising 30 minutes of standardized light activities performed in two 15-minute blocks, interspersed with 15-minute rest intervals. Furthermore, the use of fixed activity durations and rest periods enhances reproducibility and facilitates replicability in future studies. Study rigor was further strengthened by the involvement of experienced physiotherapists from Exopulse in determining the stimulation parameters, as this process requires specialized expertise. The team from Exopulse had also supported previously published studies [52,53]. In addition, the physiotherapist responsible for outcome assessment was blinded to the stimulation parameters, thereby minimizing the risk of cognitive bias and strengthening the internal validity of the study. To our knowledge, this is the first study to report spatiotemporal gait parameters with EMS use. Future controlled studies with repeated sessions should aim to confirm the efficacy of the EMS in defined patient populations. In addition, further qualitative studies of patient experience may provide insights into functional benefits in real-life activities and participation that are not fully captured by standardized quantitative measures.

### Conclusions

With an individualized approach, a single 60-minute session of EMS use may enhance functional mobility and gait quality in some participants with spasticity resulting from stroke or SCI. User qualitative feedback on EMS usability was mixed, with reported challenges in donning and doffing likely reflecting initial experiences with a novel intervention. This finding underscores the need for design enhancements and comprehensive patient onboarding and training to ensure competencies to improve user experience, support compliance, and facilitate independent use at home. Further studies are needed to validate its effectiveness. Overall, the EMS demonstrates potential as a therapeutic, nonpharmacological option for spasticity management, particularly for individuals who require accessible care beyond traditional clinical settings.

## Supplementary material

10.2196/81522 Multimedia Appendix 1 Data on upper limb function.

## Abbreviations

10MWT: 10-meter walk test
EMS: Exopulse Mollii Suit
FAC: Functional Ambulatory Category
MTS: Modified Tardieu Scale
NATU Quest: Assistive Technology Usability Questionnaire for People With Neurological Diseases
NMES: neuromuscular electrical stimulation
RI: ratio index
RMS: root mean square
SCI: spinal cord injury
sEMG: surface electromyography
TENS: transcutaneous electrical nerve stimulation
