# Supplementary material for: Clinical Outcomes of Individualized Electrostimulation Using a Wearable Electro Suit and Qualitative Feedback From a Mixed Cohort of Survivors of Stroke and Spinal Cord Injury With Spasticity: Case Series
Source: JMIR Rehabil Assist Technol. 2026 Jun 26;13:e81522. doi: 10.2196/81522 (PMC13308753; doi:10.2196/81522)
Supplement: Multimedia Appendix 1 [file rehab-v13-e81522-s001.docx]

**Appendix**

| ID | Side | Joint movement/ Antagonist muscle | sEMG index | | % Change |
| --- | --- | --- | --- | --- | --- |
|  |  |  | **Pre** | **Post** |  |
| **Stroke** | | | | | |
| 1 | Right | Elbow extension/ Biceps brachii | 75.16 | 41.59 | - 44.66 |
|  | Right | Elbow flexion/ Triceps brachii | 2.74 | 11 | 301.46 |
| 2 | Right | Elbow extension/ Biceps brachii | 78.61 | 69.97 | -10.99 |
|  | Right | Elbow flexion/ Triceps brachii | 2.82 | 4.55 | 61.35 |
| 3 | Right | Elbow extension/ Biceps brachii | 20.11 | 42.92 | 113.43 |
|  | Right | Elbow flexion/ Triceps brachii | 83.93 | 51.80 | -38.28 |
| 4 | Left | Elbow extension/ Biceps brachii | 62.88 | 53.82 | -14.41 |
|  | Left | Elbow flexion/ Triceps brachii | 6.82 | 44.10 | 546.63 |
| **Spinal Cord Injury** | | | | | |
| 5 | Right | Knee extension/ Semitendinosus and gastrocnemius | 69.74 | 57.06 | -18.18 |
|  | Right | Knee flexion/ Rectus femoris | 56.51 | 53.08 | -6.07 |
|  | Right | Ankle plantarflexion/ Tibialis Anterior | 46.31 | 48.35 | 4.41 |
|  | Right | Ankle dorsiflexion/ Gastrocnemius | 12.35 | 50.25 | 306.88 |
|  | Left | Knee extension/ Semitendinosus and gastrocnemius | 74.32 | 70 | -5.81 |
|  | Left | Knee flexion/ Rectus femoris | 57.54 | 33.4 | -41.95 |
|  | Left | Ankle plantarflexion/ Tibialis Anterior | 8.02 | 13.19 | 64.46 |
|  | Left | Ankle dorsiflexion/ Gastrocnemius | 55.85 | 92.29 | 62.25 |
| 6 | Right | Elbow extension/ Biceps brachii | 31.97 | 39.44 | 23.37 |
|  | Right | Elbow flexion/ Triceps brachii | 62.15 | 75.43 | 21.37 |
|  | Left | Elbow extension/ Biceps brachii | 19.94 | 34.97 | 75.38 |
|  | Left | Elbow flexion/ Triceps brachii | 84.02 | 85.05 | 1.23 |
| 7 | Right | Hip extension/ Rectus femoris | 70.05 | 86.40 | 23.34 |
|  | Right | Hip flexion/ Semitendinosus | 29.85 | 21.09 | -29.35 |
|  | Right | Knee extension/ Semitendinosus and gastrocnemius | 97.6 | 24.75 | -74.64 |
|  | Right | Knee flexion/Rectus femoris | 2.27 | 61.06 | 2589.87 |
|  | Left | Hip extension/Rectus femoris | 81.94 | 68.92 | -15.89 |
|  | Left | Hip flexion/Semitendinosus | 17.78 | 45.13 | 153.82 |
|  | Left | Knee extension/Semitendinosus and gastrocnemius | 35.85 | 37.54 | 4.71 |
|  | Left | Knee flexion/Rectus femoris | 64.5 | 76.84 | 19.13 |

Table 1: Pre and post sEMG index

| ID | Upper limb Fugl-Meyer Assessment | | | Box and Block Test | | | Handgrip Strength (Kg) | | |
| --- | --- | --- | --- | --- | --- | --- | --- | --- | --- |
|  | Pre | Pst | Δ | Pre | Pst | Δ | Pre | Pst | Δ |
| **Stroke** | | | | | | | | | |
| 1 | #Right: 25 | #Right: 22 | -3 | Left: 30  #Right: NT | Left: 30  #Right: NT | Left: 0 | Left: 25.2  #Right: 4.7 | Left: 26.3  #Right: 4.9 | Left: 1.1  #Right: 0.2 |
| 2 | #Right: 27 | #Right: 30 | 3 | Left: 42  #Right: NT | Left: 39  #Right: NT | Left: -3 | Left: 37  #Right: 10.2 | Left: 34.3  #Right: 10.4 | Left: -2.7  #Right: 0.2 |
| 3 | #Right: 60 | #Right: 60 | 0 | Left: 18  #Right: 7 | Left: 24  #Right: 8 | Left: 6  #Right: 1 | Left: 37.3  #Right: 26.1 | Left: 36  #Right: 23.4 | Left: 1.3  #Right: 2.7 |
| 4 | #Left: 57 | #Left: 57 | 0 | #Left: 35  Right: 15 | #Left: 42  Right: 26 | #Left: 7  Right: 1 | #Left: 15.7  Right: 49.1 | #Left: 12.6  Right: 60.1 | #Left: -3.1  Right: 11 |
| **Spinal Cord Injury** | | | | | | | | | |
| 5 | NT | NT | - | NT | NT | - | NT | NT | - |
| 6 | Left: 34  Right: 34 | Left: 51  Right: 52 | Left: 17  Right: 18 | Left: 23  Right: 18 | Left: 24  Right: 16 | Left: 1  Right: -2 | Left: 15  Right: 12.8 | Left: 16.6  Right: 11.5 | Left: 1.6  Right: -1.3 |
| 7 | NT | NT | - | NT | NT | - | NT | NT | - |

Table 2: Pre and post upper limb assessments

#Stroke affected side
